# Randomized clinical study: Partially hydrolyzed guar gum (PHGG) versus placebo in the treatment of patients with irritable bowel syndrome

**DOI:** 10.1186/s12986-016-0070-5

**Published:** 2016-02-06

**Authors:** E. Niv, A. Halak, E. Tiommny, H. Yanai, H. Strul, T. Naftali, N. Vaisman

**Affiliations:** Unit of Clinical Nutrition, Tel-Aviv Sourasky Medical Center, Sackler School of Medicine, Tel-Aviv University, Tel-Aviv, 6423906 Israel; The Department of Gastroenterology, Tel-Aviv Sourasky Medical Center, Sackler School of Medicine, Tel-Aviv University, Tel-Aviv, Israel; The Department of Gastroenterology, Sapir Medical Center, Kfar-Saba, Israel

**Keywords:** Irritable bowel syndrome (IBS), Partially hydrolyzed guar gum (PHGG), Prebiotics, Fibers

## Abstract

**Background:**

The treatment of Irritable bowel syndrome (IBS) is still challenging. Partially hydrolyzed guar gum (PHGG) is a known prebiotic fiber. To assess the effects of PHGG on clinical symptoms of IBS patients in a prospective randomized double blind placebo-controlled study.

**Methods:**

Suitable IBS patients were recruited into an 18-week-long study (2 weeks of run-in, 12 weeks of treatment and 4 weeks of follow-up). They were blindly randomized to receive 6 gr of PHGG or placebo. Treatment efficacy was evaluated by the Francis Severity IBS score, the IBS quality-of-life scores and scored parameters of weekly journal of symptoms. Deltas of changes between the final and baseline scores were compared between two groups.

**Results:**

Of 121 patients who underwent randomization, 108 patients (49 in the PHGG group and 59 in the placebo group) had all the data needed for intention-to-treat analysis. A 12-week administration of PHGG led to a significant improvement of journal bloating score in the PHGG group versus placebo (−4.1±13.4 versus −1.2±11.9, P=0.03), as well as in bloating+gasses score (−4.3±10.4 versus −1.12±10.5, *P* = 0.035). The effect lasted for at least 4 weeks after the last PHGG administration. PHGG had no effect on other journal reported IBS symptoms or on Severity and Quality of life scores. There were no significant side effects associated with PHGG ingestion. The rate of dropouts was significantly higher among patients in the placebo group compared with the PHGG group (49.15% versus 22.45%, respectively, *P* = 0.01).

**Conclusions:**

The results of this study support the administration of 6 g/day PHGG for IBS patients with bloating.

**Trial registration:**

NCT01779765

## Background

Irritable bowel syndrome (IBS) is a functional gastrointestinal disorder [1]. It is a benign, relapsing chronic disorder characterized by recurrent abdominal pain, bloating and altered bowel function. IBS has an enormous impact on the quality of life (QOL) of the patients by causing a significant disability and absence from work and school [2]. Treatment of IBS is very problematic. Despite a wide range of pharmacological and non–pharmacological therapies, there is no universally accepted therapeutic approach [3]. The etiology and underlying pathogenesis of IBS is complex and not fully understood [4]. One of the factors that contribute to IBS is abnormality in the gut flora [4]. The gut microbiota is influenced by a diverse range of factors, including diet, fiber content in food, antibiotic usage, infection, stress, and probiotic and prebiotic use [5–7].

Guar gum is a water-soluble polysaccharide found in the seeds of guar, a plant indigenous to India and Pakistan. The main component of guar gum is galactomannan. It has been used in food processing as a thickener and emulsion stabilizer. Because guar gum is extremely viscous, it is very difficult to incorporate it in food in quantities large enough to obtain a physiological effect. The proposed solution was partial hydrolyzation of guar gum. Partially hydrolyzed guar gum (PHGG) has some attractive physical and chemical properties. It is completely water soluble, invisible in solution, taste-free, stable at a low pH, heat tolerant and is known as a prebiotic fiber. Prebiotics are defined as non-digestible food ingredients that beneficially affect the host by selectively stimulating the growth and/or the activity of one bacterium or a limited number of bacteria in the colon that can improve host health. PHGG increases the concentration of bifidobacterium and lactobacilli species and increases short-chain fatty acids in the colonic lumen [8–13]. It has also been shown to have a positive effect on some medical conditions, e.g., reducing blood cholesterol and controlling blood sugar levels [14–16]. In addition, it was found to be effective in the treatment of acute diarrhea in children and adult patients of intensive care units [17–19]. PHGG has proved to be effective in softening and improving fecal output and increasing bulking capacities (fecal weight, frequency of defecation, and fecal excretory feeling) [10, 11, 20, 21] It has also been investigated as a possible treatment for IBS and found to have a positive effect, especially in constipation-predominant IBS [22–26].

Previous studies on the use of PHGG in patients with IBS, however, were biased by methodological problems (lack of placebo group and double blind randomization, small groups, short duration of study) [5, 6]. We assessed the short- and long–term effects of PHGG administration on clinical symptoms of IBS patients.

## Methods

This prospective randomized double blind placebo-controlled study was performed in the Clinical Nutrition Unit of Tel-Aviv Sourasky Medical Center (TASMC) in collaboration with the Departments of Gastroenterology in TASMC and in Sapir Medical Center, Israel. The study protocol was approved by the TASMC Helsinki committee (Number TLV-0242-12) and was registered in the NIH (NCT01779765). The patients were recruited from the medical centers’ outpatient clinics. They were screened according to the study inclusion criteria and their willingness to participate. All recruited patients provided informed consent. The inclusion criteria were as follows: (1) fulfillment of the Rome III criteria for IBS, (2) availability of at least one gastrointestinal (GI) imaging study during the last five years (colonoscopy, sigmoidoscopy, abdominal ultrasonography, barium enema) for patients older than 50 years, (3) age 18–77 years at the time of screening, (4) provision of written informed consent, and (5) commitment of availability throughout the 18-week study period. The exclusion criteria were: (1) major abdominal surgery in the past, (2) the presence of any active (organic) GI disease, (3) past or present major medical or psychiatric illness, (4) alarming symptoms (rectal bleeding, weight loss, etc.), (5) pregnancy, (6) family history of colorectal carcinoma at age younger than 50 years or a family history of IBD, (7) abnormal laboratory studies (blood biochemistry, liver enzymes, complete blood count, abnormal thyroid function, celiac serology), (8) non-adjusted diet in the case of lactose intolerance, (9) recent travel to regions with endemic parasitic diseases, (10) probiotics or prebiotics administration two weeks prior to entry into the trial and (11) antibiotic use at least 3 months prior to entry into the trial.

All types of IBS were included in the study. Based on their dominant complaint, the patients were classified as constipation-predominant, diarrhea-predominant and mixed types. According to Rome III criteria, IBS with constipation was defined as: hard or lumpy stools ≥25% and loose (mushy) or watery stools <25% of bowel movements, IBS with diarrhea--- as loose (mushy) or watery stools ≥25% and hard or lumpy stool <25% of bowel movements, Mixed IBS-- as hard or lumpy stools ≥ 25% and loose (mushy) or watery stools ≥25% of bowel movements.

The overall length of the study was 18 weeks (two weeks for the run-in period, 12 weeks of treatment and four weeks of follow-up) and was conducted between January 2013 and December 2014 (recruitment & follow up). The randomization list was made by the medical center's clinical trials pharmacy, using a block randomization of varying block size. The pharmacy staff were not involved in the conduct of the study. All of the clinical nutrition unit staff & participants were blinded during the whole trial. Patients were randomly assigned to one of two groups and received either PHGG (Sunfiber produced by Taiyo Kagaku Co., Ltd., Japan) in a dosage of three g/day for the first seven days and then six g/day for 11 weeks or placebo (Maltodextrin) in a dosage of three g/day for the first seven days and then six g/day for 11 weeks. Maltodextrin is a polysaccharide that is used as a food additive. It is produced from starch by partial hydrolysis and is usually found in the form of a white hygroscopic spray-dried powder. The patients were instructed to dissolve the sachet that contained either 6 g PHGG or placebo in a glass of water and to drink one glassful per day. Each patient was identified by a serial number, and the entire cohort underwent double blind randomization into the two groups (the study product and the placebo). They were asked not to not to take any probiotics or prebiotics during the study and to continue eating their usual diet and to take their usual medications.

A total of six visits were scheduled over the 18 weeks: the first visit at two weeks before the administration of the study product/placebo, three monthly visits during the ingestion of the study product/placebo and a follow-up visit at one month since the last ingestion of the study product/placebo. Demographic data were collected, a medical history was taken, inclusion/exclusion criteria were checked, informed consent was signed and the randomization took place at the first visit. The clinical severity of the IBS symptoms was evaluated by the Francis Severity IBS score and by the IBS quality-of-life (QOL) scores at each visit [27, 28]. In addition, the patients filled out a self-reporting daily journal of the severity of symptoms. Adverse events and compliance were monitored throughout the study period.

The Francis Severity IBS score contains five questions, each given a value from 0 (no symptoms) to 100 (most severe) for measuring the severity and frequency of IBS-associated symptoms. The total Francis Severity IBS score is a sum of all the above and ranges from 0 to 500. The QOL questionnaire consists of 34 questions, each rated from 1 (mild) to 5 (severe), and their sum yields the total QOL score, ranging from 0 to 170. This questionnaire was validated for the Hebrew language [29, 30]. The self-reporting daily journal was filled out for one week (each time at one week before the visit) and it consisted of several questions on abdominal pain, bloating, gasses and number of bowel movements per 24 h, and the score ranged from 0 to 70 for every question.

### Statistical analysis

The intention-to-treat analysis was performed on all patients who underwent randomization. An additional analysis included only those subjects who completed 12 weeks of study product/placebo administration. The primary endpoints were changes (delta) in the scores of journal parameters, the Francis Severity score, and the total QOL scores between the values at the end of treatment versus baseline and between the end of the follow-up period versus the baseline values. Comparisons of demographic and clinical variables as well as baseline severity and QOL scores between treatment and placebo groups were performed using Fisher’s exact test and the Mann–Whitney non-parametric test, as applicable. Changes in values of all scores were examined using the mixed model for analysis of variance (ANOVA with repeated measures). This model allows the evaluations of the effect of each factor on the outcome as well as the interactions between the factors. The mixed model used group (treatment versus placebo) and time as factors. A *P* value of 0.05 was taken as significant.

## Results

One-hundred and eighty-six patients underwent screening for this study. Of them, 121 patients fulfilled the inclusion criteria, signed informed consent and underwent a blind randomization into either the 6 g PHGG group or the placebo group. During the two-week run-in-period, 13 patients dropped out of the study (two patients due to travel plans, eight withdrew consent, and three were unwilling to stop probiotics). The remaining 108 patients attended the second visit of the study, provided baseline IBS parameters and started the administration of the study product/placebo. All these 108 patients were included in the intention-to-treat analysis. Table 1 presents the demographic data of these patients and their IBS baseline parameters. The PHGG group including 49 patients and the placebo group included 59 patients. The patients in both groups were of similar age (46.2±19.2 and 40.8±15.6 years for the PHGG and placebo groups, respectively, *P* = 0.1). IBS was longstanding for most of the participants, with a mean duration of 15.2±14.9 years in the study group and 13.4±12.4 years in the placebo group, *P* = 0.5. The gender distribution was similar in both groups (female predominant). Most of the patients in both groups had a mixed type of IBS (24/49 patients in the study group and 40/59 patients in the placebo group). Table 1 demonstrates the severity of IBS symptoms at baseline. The PHGG group had a more severe degree of IBS than the placebo group in terms of abdominal pain (31±15.6 versus 21.6±16.4), gasses (38.3±15.2 versus 32.5±13.5) and total severity score (307±85 versus 267.9±115.7) (*P* = 0.003, *P* = 0.036 and *P* = 0.046, respectively). Of course, the design of prospective randomized double-blind placebo controlled study could not allow us to predict this kind of distribution of patients. To overcome this, the dynamic changes in all the scores during the study were calculated as a difference from the baseline and not as an absolute value.

**Table 1:** Baseline parameters of the participants

Groups	PHGG group	Placebo group	*P* value
Number	49	59	
Age (years)	46.2 ± 19.2	40.8 ± 15.6	1.0
Gender (male/female)	15/34	22/37	
Duration of symptoms (years)	15.2 ± 14.9	13.4 ±12.4	0.5
Type of IBS			
Mixed	24	40	
Diarrhea	13	14	
Constipation	12	5	
Time from diagnosis (years)	6.2 ± 7.1	5.2 ±6.7	0.47
Journal Bloating score	32.8 ± 19.1	30.6 ± 17.6	0.6
Journal Abdominal pain score	31 ± 15.6	21.6 ± 16.4	0.003
Journal Gasses score	38.3 ± 15.2	32.5 ± 13.5	0.036
Number of bowel movements/week	13.4 ± 11	13.7 ± 9.6	0.9
Severity score	307 ± 85	267.9 ± 115.7	0.046
Quality of life score	88.8 ± 27.2	83.4 ± 26.3	0.3

Values are given in mean ± standard deviation

The patients took the study product/placebo for 12 weeks. They were then instructed to stop the administration of the products and entered four weeks of follow-up. A total of 92 patients completed one month of treatment, 78 patients completed two months, 68 patients completed three months and 41 patients completed three months of treatment and one month of follow-up. The rate of dropouts was significantly higher among patients in the placebo group (29 patients) compared to the PHGG group (11 patients) (49.15% versus 22.45%, respectively, *P* = 0.01). During the study, nine PHGG patients and nine placebo patients reported experiencing mild side effects, such as abdominal pain, gasses, diarrhea, heartburn, nausea. It was impossible to conclude if these side effects were related to PHGG because of their similarity to IBS symptoms. The side effects were mild and discontinued immediately after the stop of PHGG/placebo.

**Fig. 1:**
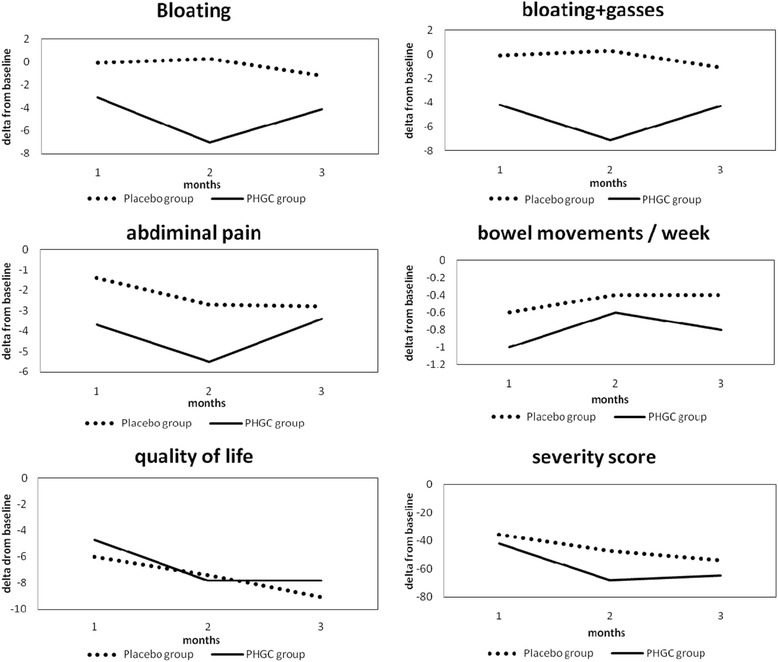
Dynamic changes in IBS parameters over 3 months of treatment expressed as delta from the baseline values

Table 2 presents the results of the effect of PHGG/placebo on IBS symptoms in our study over the 12 weeks of treatment, and Fig. 1 demonstrates the same results graphically. The assessment was performed based on intention-to-treat analysis. Delta between the change and the baseline for all the scores was chosen to be evaluated and not the absolute values of scores because the PHGG group appeared to have more severe IBS disease compared to the placebo group. The results of the study clearly demonstrated that the PHGG group had a significant improvement in bloating score (−4.1±13.4 versus −1.2±11.9) and bloating+gasses scores (−4.3±10.4 versus −1.12±10.5) compared to the placebo group (*P* = 0.03 and *P* = 0.035, respectively). This effect was consistent throughout the 12-week study period, as is apparent from the *P* values of interaction between the time and groups (*P* = 0.027 and *P* = 0.03 for bloating and bloating+gasses scores, respectively). After four weeks since the cessation of PHGG ingestion, the bloating and gasses scores started to arise again, although the difference was not significant (*P* = 0.9 and *P* = 0.83, respectively), indicating that the positive effect of PHGG did not diminish after four weeks of follow-up. Additionally, Table 2 demonstrates that PHGG had no advantage over the placebo regarding other IBS symptoms, such as abdominal pain (−3.4±11.9 versus −2.8±10.8), stool frequency (−0.8±5.1 versus −0.4±4.1), total severity score (−64.8±102.3 versus −53.9±86.7) and QOL (−7.8±20.7 versus −9.1±14.3) (*P* = 0.334, *P* = 0.713, *P* = 0.421, and *P* = 0.788, respectively). The net placebo effect resulted in a 20.1% improvement in the total severity score for the patients in the placebo group.

**Table 2:** Changes in study parameters over 3 months

Parameter	Delta 1	Delta 2	Delta 3	*P* value between groups & time	*P* value between groups
	(1st month baseline)	(2nd month baseline)	(3rd month baseline)		
Bloating score				0.027	0.03
PHGG	-3.1 ± 10.9	-7 ± 12.9	-4.1 ± 13.4		
Placebo	-0.07±9.9	0.3±11	-1.2±11.9		
Abdominal pain score				0.357	0.334
PHGG	-3.7±11.3	-5.5±14.2	-3.4±11.9		
Placebo	-1.4±10.4	-2.7±9.5	-2.8±10.8		
Bloating + gasses score				0.03	0.035
PHGG	-4.2±10.3	-7.1±10.6	-4.3±10.4		
Placebo	-0.08±6.9	0.3±10.1	-1.12±10.5		
Number of bowel movements/week				0.97	0.713
PHGG	-1±5.4	-0.6±4.6	-0.8±5.1		
Placebo	-0.6±4.9	-0.4±4.8	-0.4±4.1		
Severity score				0.441	0.421
PHGG	-41.9±88.5	-67.9±98.8	-64.8±102.3		
Placebo	-35.6±74.0	-47.3±76.0	-53.9±86.7		
Quality of life score				0.643	0.788
PHGG	-4.7±13.6	-7.8±17.6	-7.8±20.7		
Placebo	-6.0±11.8	-7.4±12.8	-7.4±12.8

Because the score of stool frequency has different meanings in patients with diarrhea-predominant and constipation-predominant IBS, the analysis of this score was performed separately for the patients with these two disease types. PHGG intake did not decrease the stool frequency in diarrhea-predominant patients nor did it increase stool frequency in constipation-predominant patients compared to the placebo group. However, a proper statistical evaluation could not been performed because of the relatively small number of patients in the groups with these types of IBS.

## Discussion

This prospective, randomized, double blind placebo-controlled study on the effects of 12 weeks of administration of 6 g/day PHGG on the clinical symptoms of IBS patients revealed that PHGG produced a significant improvement on bloating and gasses symptoms of IBS patients compared to placebo. Its use was not associated with any significant side effects. The effect of this prebiotic lasted at least four weeks after the cessation of its ingestion. PHGG did not, however, have any effect on abdominal pain, stool frequency, or QOL. Therefore, the results of this study support the administration of 6 g/day PHGG for IBS patients with bloating and gasses.

The administration of PHGG in IBS patients has been reported by others. Giaccari et al [24] administered PHGG to 134 IBS patients in addition to a balanced diet and found a positive effect of PHGG on various IBS symptoms. Their study, however, was observational and not randomized, there was no placebo group, and PHGG was taken as a supplementation to a balanced diet. Paresi et al [22] treated 188 IBS patients with a high–fiber diet supplementation (30 g/day of wheat bran) or PHGG (5 g/day) for 12 weeks. Improvements in core IBS symptoms were observed with both the bran and PHGG, but the latter was better tolerated and preferred by patients. That crossover study was very interesting, but the lack of a placebo group and a follow-up period posed important limitations. The same authors [23] published an important paper in which they compared the effects of PHGG administered at two dosages (5 and 10 g/day) for 3 months in patients with IBS, followed by three months of follow-up. The symptoms of IBS and QOL improved significantly for the patients in both dosage groups. However, this positive effect was significantly reduced during the three-month follow-up.

This study’s major strength lies in its being the first prospective, randomized, double blind placebo-controlled investigation of the effect of PHGG in the setting of IBS. The profound placebo effect on IBS symptoms (ranging from 20-50%) is well-established [5, 6]. In the current study, the net placebo effect was about 20%. In fact, every significant study regarding IBS should be placebo-controlled. Therefore a proven statistically significant effect on bloating and gasses over placebo in our study has an important significance. One weakness of the current study is the relatively high dropout rate, although most of the dropouts were in the placebo group (47.5%) compared with the PHGG group (23.5%), supporting a positive effect of PHGG. The follow-up period in our study was four weeks, which is adequate, but a longer period would be able to establish whether PHGG treatment should be episodic or continuous.

This study did not focused on the mechanism of PHGG influence on the GI tract of IBS patients. Previous studies proved an ability of PHGG to act as a prebiotic fiber and influence the microbiota. For example, in two clinical studies (one –in constipated women and the second one in healthy volunteers) several weeks of PHGG administration resulted in the increase of Bifidobacterium and Lactobacillus spp and the decrease of Clostridium spp, Enterobacteriaceae and Streprococcaceae [9, 11]. Moreover, in vitro fermentation of PHGG results in an active production of short chain fatty acids, which are important for colon health as a primary energy source of colonocytes [31]. Therefore, presumably the mechanisms of PHGG impact on the GI tract of IBS patients are through a microbiota change and a positive stimulating effect on colonocytes. However, the exact mechanism of PHGG influence is still unknown and should be a subject of further research.

## Conclusion

This prospective, randomized, double blind placebo-controlled study supports the administration of 6 g/day PHGG for IBS patients with an expected clinical effect on bloating and gasses. It demonstrates that prebiotic administration has a significant effect on the symptoms of such challenging clinical condition as IBS. This finding emphasizes the significance of gut microbiota in pathogenesis and treatment of IBS.

## Abbreviations

IBS: Irritable bowel syndrome
PHGG: Partially hydrolyzed guar gum
QOL: Quality of life
GI: Gastrointestinal
IBD: Inflammatory Bowel Syndrome
